# The utility of FDG-PET for assessing outcomes in oligometastatic cancer patients treated with stereotactic body radiotherapy: a cohort study

**DOI:** 10.1186/1748-717X-7-216

**Published:** 2012-12-18

**Authors:** Abhishek A Solanki, Ralph R Weichselbaum, Daniel Appelbaum, Karl Farrey, Kamil M Yenice, Steven J Chmura, Joseph K Salama

**Affiliations:** 1 Department of Radiation and Cellular Oncology, University of Chicago Medical Center, 5758 South Maryland Ave., MC 9006, Chicago, IL 60637, US; 2 Cancer Research Center, University of Chicago, Chicago, IL, US; 3 Ludwig Center for Metastasis Research, University of Chicago, Chicago, IL, US; 4 Department of Radiology, University of Chicago Medical Center, 5841 South Maryland Ave., MC 2026, Chicago, IL 60637, US; 5 Department of Radiation Oncology, Duke University Medical Center, Box 3085, Durham, NC 27713, USA

**Keywords:** “Stereotactic body radiotherapy”, SBRT, oligometastasis, “PET response”

## Abstract

**Background:**

Studies suggest that patients with metastases limited in number and destination organ benefit from metastasis-directed therapy. Stereotactic body radiotherapy (SBRT) is commonly used for metastasis directed therapy in this group. However, the characterization of PET response following SBRT is unknown in this population. We analyzed our cohort of patients to describe the PET response following SBRT.

**Methods:**

Patients enrolled on a prospective dose escalation trial of SBRT to all known sites of metastatic disease were reviewed to select patients with pre- and post-therapy PET scans. Response to SBRT was characterized on PET imaging based on standard PET response criteria and compared to CT based RECIST criteria for each treated lesion.

**Results:**

31 patients had PET and CT data available before and after treatment for analysis in this study. In total, 58 lesions were treated (19 lung, 11 osseous, 11 nodal, 9 liver, 6 adrenal and 2 soft tissue metastases). Median follow-up was 14 months (range: 3–41). Median time to first post-therapy PET was 1.2 months (range; 0.5-4.1). On initial post-therapy PET evaluation, 96% (56/58) of treated metastases responded to therapy. 60% (35/58) had a complete response (CR) on PET and 36% (21/58) had a partial response (PR). Of 22 patients with stable disease (SD) on initial CT scan, 13 had CR on PET, 8 had PR, and one had SD. Of 21 metastases with PET PR, 38% became CR, 52% remained PR, and 10% had progressive disease on follow-up PET. 10/35 lesions (29%) with an initial PET CR progressed on follow-up PET scan with median time to progression of 4.11 months (range: 2.75-9.56). Higher radiation dose correlated with long-term PET response.

**Conclusions:**

PET response to SBRT enables characterization of metastatic response in tumors non-measurable by CT. Increasing radiation dose is associated with prolonged complete response on PET.

## Background

Metastases are the most common cause of cancer related mortality. The standard treatment for most metastatic cancer patients is usually chemotherapy or hormonal deprivation. Although chemotherapy has improved median survival duration in patients with metastatic cancer, response rates are low and chemotherapy is rarely curative, except in cases of hematologic malignancies and germ cell tumors [1-6]. Previously, it has been proposed that within the spectrum of metastatic disease, a group of patients exists with metastases limited in number and location (i.e. oligometastatic patients), who may benefit from metastasis-directed therapy [7]. This hypothetical disease state is supported by long-term survival rates of 20-50% in patients undergoing limited pulmonary and hepatic metastasectomy [8,9]. Recent technical developments in radiotherapy planning and delivery have allowed metastatic patients who are not medically fit for surgery, or those who are technically unresectable, to be treated with a few fractions of high doses of radiation to each lesion. Termed stereotactic body radiotherapy (SBRT), these techniques have been shown in prospective series to render some patients with limited metastases disease-free [10,11] .

Response assessment following SBRT is typically performed via computed tomography (CT) scans with each individual lesion measured using RECIST criteria [12]. However, due to architectural changes of normal tissues surrounding treated metastases, particularly post-SBRT scarring, RECIST criteria are often difficult to use [13]. Furthermore, osseous metastases are not measureable by standard RECIST criteria, complicating assessment of treatment response in these regions commonly involved with oligometastases.

2-[18F]-fluoro-2-deoxy-D-glucose positron emission tomography (PET) imaging is a common metabolic imaging technique used to stage many cancer patients as well as assess outcomes following treatment. PET is particularly helpful in cases where anatomy has been altered, such as due to radiotherapy fibrosis or necrosis, where magnetic resonance imaging (MRI) and computed tomography (CT) may not be able to differentiate malignant pathology from post treatment changes [14,15]. To our knowledge, there are no data on the use of PET for assessing SBRT response in patients with oligometastatic disease. Therefore, we evaluated the utility of PET as an indicator of treatment response in oligometastatic patients undergoing SBRT.

## Methods

Patients included in this analysis were selected from participants in an IRB approved dose escalation study assessing the role of SBRT to all known sites of metastases in oligometastatic patients. From these patients, we identified those with PET imaging both prior to and after radiotherapy to form our study population.

We have previously reported trial methods and treatment outcomes [10]. Briefly, patients were eligible if they had pathologically confirmed stage IV metastatic cancer, were ≥ 18 years of age, had life expectancy of > 3 months, with metastases amenable to radiation therapy as seen on standard imaging (CT, MRI, PET, or bone scan). Patients were excluded if they had coexisting malignancies, uncontrolled medical comorbidity, active infectious processes, or exudative, bloody, or cytologically malignant effusions. Concurrent systemic chemotherapy was not allowed during radiotherapy. Each metastatic lesion was grouped into one of the following five disease sites: liver, lung, abdominal, head and neck, or extremity.

### Radiotherapy

Prior to SBRT, all patients were simulated in custom-made immobilization devices. Typically, patients underwent contrast-enhanced CT-based radiotherapy treatment planning, including four-dimensional CT scans as needed to allow visualization and assessment of internal organ motion. For tumors with motion, an internal target volume (ITV) was generated based on maximal intensity projection images from either the entire respiratory cycle or end-expiratory phase, as appropriate based on tumor motion. Otherwise, the gross tumor volume (GTV) was contoured on free breathing scans. Diagnostic images (MRI, PET, triple-phase CT, etc.) were anatomically registered to the planning CT scans to assist with target delineation. GTVs were delineated on each CT slice, typically with a 5–7 mm expansion, for organ motion and set-up uncertainty only, to create the PTV. For lesions within the bone, a larger CTV expansion was used to encompass microscopic bony involvement.

3D conformal techniques, including both coplanar and non-coplanar beam arrangements were used for radiation planning. Radiotherapy was typically prescribed to the 85% isodose line. The starting dose for all sites was 24 Gy in 3 fractions. A standard 3 + 3 dose escalation schema was used with SBRT dose per treatment increasing in 2 Gray (Gy) increments to each lesion. Each lesion received the same dose throughout all three treatments, but different lesions within the same patient could receive different doses. The time between consecutive fractions was a minimum of 48 hours and a maximum of 192 hours. At the discretion of the treating radiation oncologist, alternative dosing schedules could be used for larger metastases not amenable to three fraction treatment as this had been previously shown to be safe and effective for metastatic patients [16].

Regardless of dose and fractionation used, prior to each treatment, image-guidance with gated kilovoltage (KV) orthogonal images and/or non-gated KV cone beam CT scans were acquired and appropriate adjustments made by the attending physician. With bony anatomy correlated, each metastasis was verified to fall within the planning target volume. If this did not occur, patients were replanned.

### Imaging schedule

Baseline CT scans of the chest, abdomen, and pelvis along with PET scans were obtained no more than 1 month prior to the start of treatment. Patients returned to clinic every 2 weeks for the first month following treatment, then monthly for the first 3 months, followed by every 3 months thereafter. Imaging was performed one month following the completion of SBRT and then prior to each additional follow-up visit, at which time a complete history and physical was also performed. All images were evaluated by radiologists at the University of Chicago Medical Center.

### Response assessment

Each metastatic lesion was independently considered a target lesion and assessed for response to its localized treatment dose as described below. All target and non-target lesions were followed on subsequent imaging for analysis of patterns of progression.

CT response was defined by RECIST criteria [12]. Based on these criteria, a complete response (CR) was total resolution of tumor, partial response (PR) was ≥ 30% decrease in the longest diameter of a lesion, stable disease (SD) was between a < 30% decrease and a < 20% increase in the longest diameter, and progressive disease (PD) was a ≥ 20% increase in the lesion.

PET scan interpretation was performed by a nuclear medicine radiologist. Positive PET uptake was determined by visual analysis and/or maximum standardized uptake value (SUV) > 2.5. Areas of no uptake or diffuse poorly defined low uptake were considered to be non-malignant and negative. A PET CR was defined as complete resolution of uptake or normalization to surrounding tissue uptake. PET PR was a ≥ 30% decrease in maximum SUV or partial decrease in uptake by visual assessment. PET SD was no change in uptake by visual assessment or < 30% decrease or < 20% increase in maximum SUV. Progressive disease was defined as increased uptake by visual assessment of ≥ 20% increase in SUV.

## Results

This analysis was performed after the first 50 patients participating in the dose escalation trial had been enrolled. Of these, 31 patients had PET and CT data available before and after treatment for analysis in this study. Patient and tumor characteristics are presented in Table 1. Median follow-up was 14 months (range: 3–41 months). Median time from cancer diagnosis to the development of metastastic disease was 11.1 months (range: 0–299 months). Median time from development of metastatic disease to SBRT was 6.9 months (range: 0–59.1 months). Median number of chemotherapy regimens received prior to SBRT was 1 (range: 0–4). 

**Table 1:** Patient and Tumor Characteristics.

**Characteristic**	**N**	**%**
Total Patients	31	100%
Patient sex		
Male	13	42%
Women	18	58%
Primary Site		
Non-small Cell Lung	9	16%
Sarcoma	5	9%
Breast	4	7%
Head and Neck	4	7%
Colon	4	7%
Small Cell Lung	3	5%
Basal Cell	1	2%
Ovarian	1	2%
Metastatic Sites		
Lung	19	33%
Lymph Node	11	19%
Osseous	11	19%
Liver	9	16%
Adrenal	6	10%
Soft tissue	2	3%
Dose		
24 Gray	19	33%
30 Gray	20	35%
36 Gray	8	14%
42 Gray	9	16%
50 Gray	2	3%

The majority of patients were women (52%). The most common primary histology treated was non-small cell lung cancer (9 patients), followed by sarcoma (5 patients), breast (4 patients), colon (4 patients), and head and neck cancer (4 patients). In total 58 metastatic lesions were treated, including 19 lung, 11 osseous, 11 nodal, 9 liver, 6 adrenal and 2 soft tissue. Treatment dose was selected per protocol and listed in Table 1. Two patients were treated with 50 Gy in 10 daily fractions of 5 Gy.

Following treatment, the median time to the first post-therapy PET was 1.2 months (range: 0.5-4.1). All patients had one post-therapy PET, 24 of 31 (77%) had two post therapy PET scans and 9 of 31 (29%) had three post-therapy PET scans. The median time to the second PET scan was 4.4 months (range: 1.4-26.8 months) and the median time to third PET scan was 7.1 months (range: 6.4-20.4 months).

### Initial response to therapy

On initial post-therapy PET evaluation, 96% (56 of 58) of treated metastases responded to therapy. This included 60% (35 of 58) with a PET CR and 36% (21 of 58) with a PET PR. Forty-eight of 58 treated tumors were evaluable for CT response. The other ten lesions were non-measurable by RECIST criteria. On initial post-therapy CT evaluation, 52% (25 of 48) had a response, with 19% (9 of 48) being a CR and 33% (16 of 48) a PR. Initial CT response to therapy was generally in agreement with PET response. Eight of nine metastases with an initial CR on CT also had an initial PET CR. One patient with an adrenal metastasis had resolution of his adrenal metastasis on CT scan but persistent activity (SD) on PET scan. Of the ten patients with non-measureable disease on initial CT scan, all had a PET response with 5 having a PET CR and 5 with a PET PR. Of the 22 patients with SD on initial CT scan, 13 had a PET CR, 8 had a PET PR, and one had PET SD, suggesting that PET was useful in determining initial response in patients with stable disease on initial CT scan.

Patients with adrenal metastases and nodal metastases were more likely to have an initial PET CR than other metastatic sites. Eighty-three percent (5 of 6) of adrenal lesions and 82% (9 of 11) of nodal lesions had an initial PET CR. In comparison, 53% (9 of 19) of pulmonary metastases, 56% (5 of 9) of hepatic metastases, and 55% (6 of 11) of osseous metastases had an initial PET CR.

Increasing radiation dose was not associated with increasing initial PET CR as shown in Table 2. Forty-eight percent of patients treated at 24 Gy, 60% treated at 30 Gy, 50% treated at 36 Gy, and 55% treated at 42 Gy had an initial PET CR. All patients treated with a minimum of 36 Gy had an initial PET response. No patient treated with 50 Gy in 5 Gy fractions had a PET CR, but only two patients were treated with this regimen.

**Table 2:** Comparison of PET and CT response by dose cohort.

**Dose**	**Initial Response**								
	**CR**		**PR**		**SD**		**PD**		**Non-measurable**
	**CT**	**PET**	**CT**	**PET**	**CT**	**PET**	**CT**	**PET**	**CT**
	**n (%)**	**n (%)**	**n (%)**	**n (%)**	**n (%)**	**n (%)**	**n (%)**	**n (%)**	**n (%)**
24 Gy (n=19)	4 (24%)	14 (74%)	8 (47%)	4 (21%)	4 (24%)	0	1 (6%)	1 (5%)	2 (11%)
30 Gy (n=20)	3 (21%)	12 (60%)	3 (21%)	7 (35%)	8 (57%)	1 (5%)	0	0	6 (30%)
36 Gy (n=8)	1 (14%)	4 (50%)	1 (14%)	4 (50%)	5 (71%)	0	0	0	1 (13%)
42 Gy (n=9)	1 (14%)	5 (55%)	2 (29%)	4 (44%)	5 (71%)	0	0	0	1 (13%)
50 Gy (n=2)	0	0	2 (100%)	2 (100%)	0	0	0	0	0
Total	9 (19%)	35 (60%)	16 (33%)	21 (36%)	22 (46%)	1 (2%)	1 (2%)	1 (2%)	10 (17%)

*CR* complete response.

*Gy* gray.

*PR* partial response.

*PD* progressive disease.

*SD* stable disease.

### Utility of continued PET surveillance

Continued PET follow-up was useful. Table 3 depicts the initial and cumulative PET response of each treated lesion. Of the 21 metastases with an initial PET PR, 8 (38%) became a PET CR, 11 (52%) remained a PET PR, and 2 (10%) demonstrated PD on PET scan. Thus, over half of patients with a PET PR maintained the PR and over one third improved to a PET CR. Similarly, 12 of 35 lesions (35%) with an initial PET CR progressed on follow-up PET scan. One lesion with an initial SD on PET, converted to a CR with further PET imaging. The median time to conversion from CR to PD was 4.11 months (range: 2.75-9.56) and corresponded with the second PET scan.

**Table 3:** Initial and Cumulative PET Response per treated metastatic lesion.

**Initial Response**	**Cumulative Response**				
	**CR**	**PR**	**SD**	**PD**	**Total**
CR	22 (63%)	1 (3%)	0	12 (35%)	35
PR	8 (38%)	11 (52%)	1 (5%)	1 (5%)	21
SD	1 (100%)	0	0	0	1
PD	0	0	0	1 (100%)	1
Total	31 (53%)	12 (21%)	1 (2%)	14 (24%)	58

*CR* complete response.

*PR* partial response.

*SD* stable disease.

*PD* progressive disease.

Contrary to initial PET response, long-term PET response correlated with increasing radiotherapy dose. All lesions receiving >30 Gy had responded on the initial post-therapy PET. Long term metabolic response was maintained with higher radiotherapy doses. Of patients with an initial PET CR, 50% (7 of 14) progressed metabolically at the 24 Gy level, compared to 16.6% (2 of 12) at 30 Gy, 25% (1 of 4) at 36 Gy, and 0% (0 of 5) at 42 Gy.

Long-term PET response also differed based on metastasis location. Of the 19 pulmonary metastases, 9 (47%) had a long-term PET CR and 6 (32%) had a PET PR. Seven of eleven (63%) lymph node metastases had long term PET CR. Six of nine hepatic metastases remained in metabolic response long-term as well. All osseous metastases had either a CR or PR on initial PET, and none progressed by metabolic imaging at last follow-up.

Certain primary histologies were associated with increased risk of progression on PET. Three of four (75%) metastatic lesions from small cell lung cancer progressed metabolically. Additionally, four of seven (57%) metastatic colon cancer lesions had metabolic progression. All breast (5 of 5), sarcoma (7 of 7), and four of five head and neck cancer metastases remained in metabolic response at last follow-up.

Follow-up PET imaging was also useful in the detection of further metastatic progression outside of treated sites. Distant progression was detected on PET scans in 17 of 31 patients (55%). PET was able to identify radiographically occult distant metastases in 5 of 31(16%). Median time to progression after SBRT was 4.5 months (range: <1 month-35.3 months). At 6 months, 45% of patients were free of progression, while at one year, 39% were free of disease. There was no difference in the pattern of progression (treated metastasis vs. new metastatic site) based on histology (p=0.06), or number of metastases at progression (p=0.87). There were two few numbers of some histologies to compare the time to progression. 8 patients had progression in treated metastases as the first site of progression while 15 had progression in new metastases and 2 had both progression in treated and new metastatic sites. 52 patients had 1 site of progression, 2 patients had 2 sites, 5 patients had 3 sites, and one each had 5 and 6 sites. Median survival was 31.7 months (range: 5.9 months-55.1 months) in our cohort of patients.

## Discussion

Clinical decision making for metastatic patients undergoing treatment hinges on the clinician’s ability to accurately assess the initial therapy response, as well as the duration of response. CT and MRI are routinely used to evaluate response to therapy, with RECIST criteria being used to assess response for patients on study and similar criteria used for off study patients. However, as used in routine clinical practice, these technologies and measurement standards have limitations. Postsurgical and postradiotherapy changes alter fascial planes and create fibrosis and necrosis of both malignant tissue as well as surrounding normal tissue, often making it difficult to differentiate between malignant and non-malignant tissues in surveillance CT and MRI imaging [14,15,17,18]. This is especially the case when the size of tumor does not change even though pathologically the tissue is no longer malignant.

PET technology is reliant upon the altered metabolic biology of malignant cells compared to normal cells [19]. 2-[18F]-fluoro-2-deoxy-D-glucose is the most frequently utilized radiotracer in clinical practice. As availability and technology improve, PET is increasingly being used in diagnosis, staging, radiotherapy planning, early response assessment, and restaging after completion of therapy for several malignancies [19]. In the detection of recurrent head and neck tumors, PET has been shown to have a sensitivity and specificity approaching 90-100% [20-22], and in primary lung cancer, PET is increasingly used to differentiate tumor from atelectasis and consolidation.

**Figure 1:**
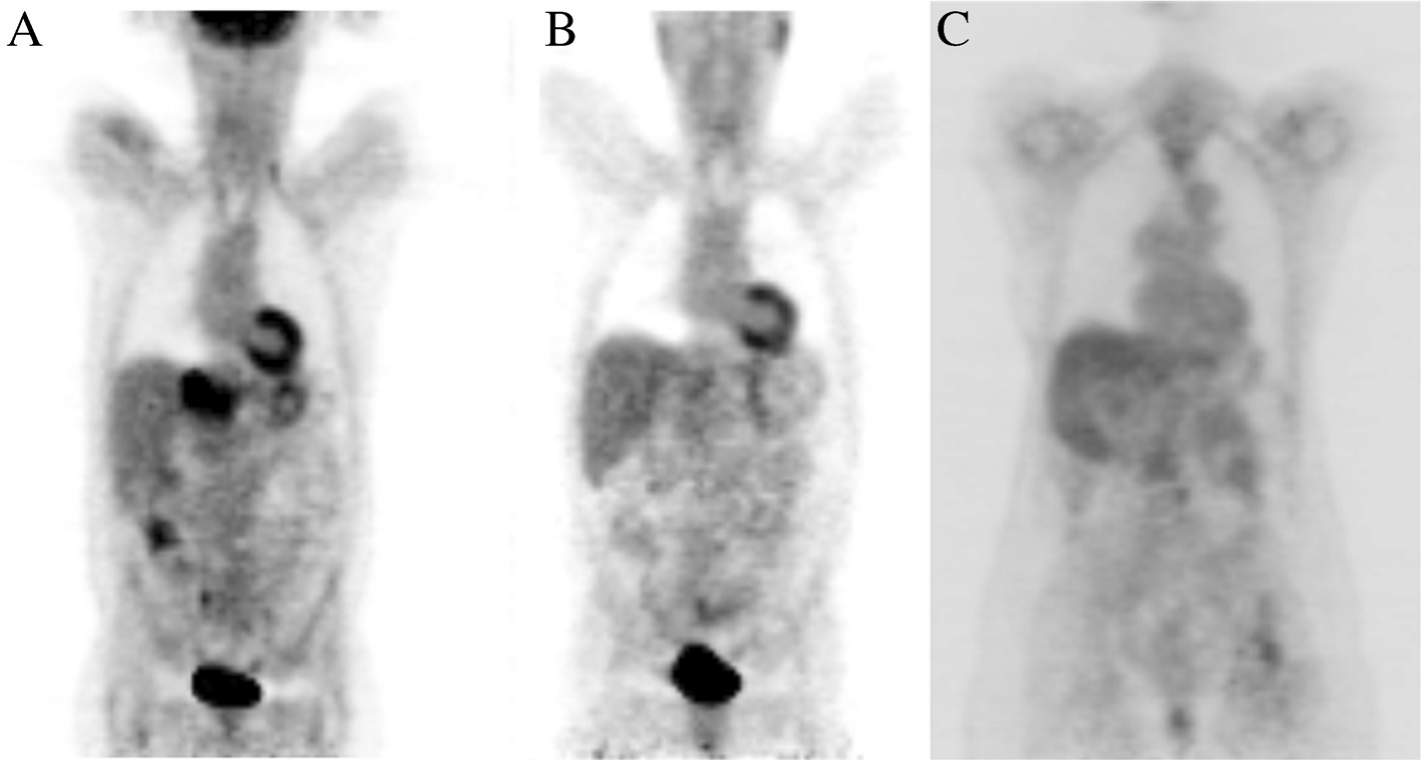
**Example PET response.** Coronal images of a PET scan of a patient prior to SBRT (**A**). Notice the hypermetabolic lesion in the liver has had a complete response at 1 month following therapy (**B**), and continues to have a complete response at 19 months post-therapy (**C**).

In our study, we found that FDG-PET appeared to be a useful adjunct for response assessment for patients with oligometastatic disease treated with SBRT. Not only did most patients with a CT response have a PET response, but patients who were not evaluable with CT were able to be assessed with PET scan effectively. Furthermore, many of the patients with partial response on PET proceeded to have a complete PET response with further follow-up. This can be seen in Figure 1, which depicts the sequential PET imaging from a patient in our study.

In our patient population, PET was helpful in assessing a response in patients with stable disease by anatomic evaluation on CT. This has significant implications on clinical decision making. The concern for treatment resistant disease makes it crucial for the clinician to rely on imaging to monitor disease response. However, disease may not morphologically change on CT scan. In this situation, PET allows for an understanding of tumor physiology, and hence a decrease in metabolic uptake (PR or CR) would indicate decreased tumor activity and possible response to treatment. In the setting of a stable CT examination, this could decrease unnecessary or untimely changes of treatment for suspected resistance to treatment. A stable CT scan can mean a number of things in this situation. The patient could have fibrosis or necrosis secondary to treatment, or conditions such as atelectasis in the lung [14,22,23]. On CT it could be difficult to identify viable tumor tissue compared to these post-treatment changes, but on PET it would be easier to distinguish tumor from normal tissue. PET may have a role in assessing response after SBRT in primary and metastatic lung tumors [24, 25, 26]. PET imaging’s strength is in this ability to differentiate for both planning purposes as well as long term analysis of patterns of progression.

**Figure 2:**
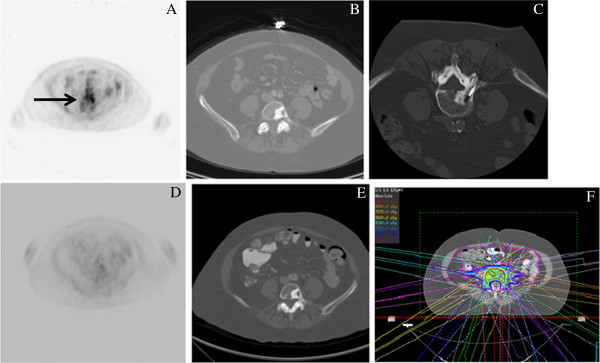
**PET response in a lesion non-measurable by CT.** Pretreatment PET (**A**) and CT (**B**) of a patient with a bony metastatic lesion. The black arrow points to the area of PET avidity on the pretreatment PET (**A**). PET (**D**) and CT (**E**) 1 month after completion of SBRT shows what seems to be stable disease (SD) on CT, but RECIST criteria cannot be used for bony lesions. Post treatment PET shows almost complete response (CR). Pretreatment biopsy (**C**) and SBRT plan (**F**) are also depicted.

Interestingly, patients in our study with osseous metastases had very encouraging early and late response to SBRT. As osseous metastases cannot be evaluated with standard RECIST criteria, the use of PET in response of assessment is particularly important [12]. As is demonstrated by Figure 2, PET is an effective tool to use in evaluating for response in these patients.

This is the first study to evaluate the utility of PET for assessing response following SBRT for patients with limited metastatic disease. PET has previously been evaluated in patients treated with SBRT for Stage 1 non-small cell lung cancer. Ishimori et al. studied the use of FDG-PET in assessing the response of patients treated with SBRT [18]. PET studies were performed 1 week before and 1–8 weeks after treatment. Of nine patients with imaging, two had a PET CR and seven had a PET PR. Hoopes et al. found that in 28 patients with PET follow-up after SBRT, 4 patients had prolonged elevation of SUV at 22–26 months but no clinical or radiographic evidence of local, nodal, or distant disease [11,23].

It is interesting to note that following treatment, residual PET activity may be present. This could indicate a protracted inflammatory response to higher dose per fraction radiotherapy. While protracted low level PET activity has not been seen to predict for recurrence or late toxicity, this may warrant further investigation.

It is important to acknowledge that a limitation of our study is the relatively short median follow-up of our cohort, mostly due to death from malignancy. Additionally, it is difficult to make conclusions from our data regarding the cumulative PET and CT response to SBRT for time periods longer than our follow-up. Our results must also be interpreted in light of the small and heterogeneous patient population as well as the non-standardized follow-up PET schedule amongst our cohort.

## Conclusions

PET was effective at detecting responses in oligometastatic patients undergoing curative intent SBRT. CR by CT correlated with PET CR. PET was able to detect response in patients with stable disease by RECIST criteria. Long-term PET response was related to radiation dose delivered. PET scan should be part of the routine follow-up for patients undergoing curative intent SBRT to determine response to therapy.

Supported by the Ludwig Center for Metastasis Research and the University of Chicago Cancer Research Center Grant 5–30073

## Competing interests

The authors declare that they have no competing interests.

## Authors’ contributions

AS reviewed charts and imaging reports of patients in the current study, updated the database, performed analysis, and drafted manuscript and figures/tables. JS was primary investigator of trial that the current study’s patients were treated on, primary oncologist for patients on study, participated in database analysis as well as creating and critically revising the manuscript. KY and KF provided physics support for patients treated on trial and critically reviewed and revised manuscript. DA evaluated PET imaging of patients in current study and critically reviewed manuscript. RW and SC participated in creating trial in which patients were treated, and critically evaluated the manuscript. All authors read and approved the final manuscript.
